# Turntable Paper-Based Device to Detect *Escherichia coli*

**DOI:** 10.3390/mi12020194

**Published:** 2021-02-13

**Authors:** Yung-Chih Wang, Yao-Hung Tsai, Ching-Fen Shen, Ming-Yao He, Yi-Chen Fu, Chen-Yu Sang, Yi-Tzu Lee, Chao-Min Cheng

**Affiliations:** 1 National Defense Medical Center, Division of Infectious Diseases and Tropical Medicine, Department of Internal Medicine, Tri-Service General Hospital, Taipei 114, Taiwan; wystwyst@gmail.com; 2 Institute of Biomedical Engineering, National Tsing Hua University, Hsinchu 300, Taiwan; michaeltsai45@gmail.com (Y.-H.T.); martin880210@gmail.com (M.-Y.H.); sandy216621@gmail.com (Y.-C.F.); sang0205@gmail.com (C.-Y.S.); 3 Department of Pediatrics, National Cheng Kung University Hospital, College of Medicine, National Cheng Kung University, Tainan 701,Taiwan; drshen1112@gmail.com; 4 Department of Emergency Medicine, Taipei Veterans General Hospital, Taipei 112, Taiwan; 5 Faculty of Medicine, School of Medicine, National Yang-Ming University, Taipei 112, Taiwan

**Keywords:** *Escherichia coli*, paper-based ELISA, point-of-care, turntable

## Abstract

*Escherichia coli* has been known to cause a variety of infectious diseases. The conventional enzyme-linked immunosorbent assay (ELISA) is a well-known method widely used to diagnose a variety of infectious diseases. This method is expensive and requires considerable time and effort to conduct and complete multiple integral steps. We previously proposed the use of paper-based ELISA to rapidly detect the presence of *E. coli*. This approach has demonstrated utility for point-of-care (POC) urinary tract infection diagnoses. Paper-based ELISA, while advantageous, still requires the execution of several procedural steps. Here, we discuss the design and experimental implementation of a turntable paper-based device to simplify the paper-based ELISA protocols for the detection of *E. coli*. In this process, antibodies or reagents are preloaded onto zones of a paper-based device and allowed to dry before use. We successfully used this device to detect *E. coli* with a detection limit of 10^5^ colony-forming units (colony-forming unit [CFU])/mL.

## 1. Introduction

*Escherichia coli*, a Gram-negative bacterium commonly found in the intestinal flora of humans and animals, is the etiological agent responsible for a variety of common infectious diseases, including urinary tract infections [1] and gastroenteritis [2]. There is a need for rapid, timely, and specific diagnostic procedures to detect *E. coli* infection. This is especially true considering emerging drug resistance issues plaguing healthcare systems worldwide [3,4]. Timely diagnosis plays a key role in optimal disease management, because appropriate early antibiotic therapy can decrease septic patient mortality [5].

It takes a long time to identify the pathogen that causes infectious diseases using traditional methods [6]. A point-of-care (POC) diagnostic tool can provide timely, convenient, and appropriate information for those in need of medical care [7]. Several rapid molecular-based methods for pathogen identification have been developed and are currently in broad clinical use [7,8,9]. However, their high cost and instrument dependency restricts their use in resource-limited areas [7].

The enzyme-linked immunosorbent assay (ELISA) is an appropriate and highly sensitive method for quantifying antigen content in a short timespan (h). While ELISA has been used as a convenient tool for diagnosing infectious diseases [10,11], it has some disadvantages: it is labor-intensive, requires costly antibody preparation, and multiple steps must be completed to produce results [12]. Previously, we developed a paper-based ELISA tool to rapidly detect the presence of *E. coli*. This tool demonstrated promising performance, providing results within 5 h with a detection limitation of 10^5^ cells/mL, and was considered to be a suitable tool for diagnosing urinary tract infection [13]. As this methodology relies on ELISA techniques, it also requires the completion of several steps, as well as the addition of various antibodies or reagents, to produce results. In this study, we aimed to use a turntable paper-based device for the rapid detection of *E. coli* that relies on the preloading of antibodies or reagents in order to simplify the ELISA process.

## 2. Materials and Methods

### 2.1. Bacterial Suspensions Preparation

We used the *E. coli* DH5α isolate in the study. The *E. coli* DH5α were cultured overnight at 37 °C in 5 mL of Luria-Bertani (LB) broth, with shaking at 250 rpm. We then added bacterial suspension aliquots to fresh LB broth and cultured with shaking at 250 rpm at 37 °C to reach the mid-logarithmic growth phase. The bacterial cell concentration was assessed by measuring optical density of a culture sample at 600 nm (OD_600_) using a Nanodrop spectrophotometer (Thermo Fisher Scientific; Waltham, MA, USA). The bacterial samples were then serial-diluted in LB broth and plated onto Tryptic Soy Agar (TSA) plates for viable cell counting after culturing overnight at 37 °C. Bacterial samples (10^8^ colony-forming units [CFU]/mL) were subsequently serially diluted with LB medium to produce seven different sample concentrations: 0 CFU/mL, 10^3^ CFU/mL, 10^4^ CFU/mL, 10^5^ CFU/mL, 10^6^ CFU/mL, 10^7^ CFU/mL, and 10^8^ CFU/mL. Samples without cells were used as the control.

### 2.2. Paper-Based ELISA

**Figure 1:**
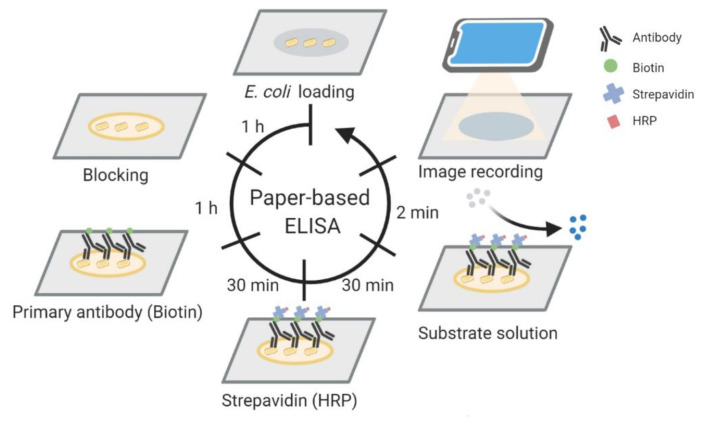
Schematic illustration of a paper-based enzyme-linked immunosorbent assay (ELISA) for *Escherichia coli* detection. *E. coli* suspensions were loaded onto the paper, the paper was dried for one hour, and blocking was completed by applying 1% BSA for one hour. Anti-*E. coli* biotin conjugate antibody and horseradish peroxidase (HRP)-conjugated streptavidin were applied for 30 min each. Substrate solution was added, and the colorimetric results were photographed using a smartphone after 2 min.

As mentioned, we previously used paper-based ELISA to detect the presence of *E. coli*. [13]. The elements of this process are as follows. We applied bacterial suspension samples, dropwise, onto Whatman^®^ Fusion 5 paper (Sigma–Aldrich; St. Louis, MO, USA) and allowed the paper to dry for an hour. Next, we added 200 μL of 1% bovine serum albumin (BSA) blocking reagent and waited an hour. After blocking, we added the anti-*E. coli* biotin conjugate (Applied Biological Materials [ABM] inc.; Richmond, British Columbia, Canada) antibody diluted 1:1000 with 1% BSA and waited for 30 min. We subsequently washed the sample target zones with 200 μL of 0.05% Tween 20 in phosphate-buffered saline (PBST) and then added 200 μL of horseradish peroxidase (HRP)-conjugated streptavidin (Abcam, Cambridge, UK) and waited for 30 min to complete the reaction. We then washed the sample target zones 4 times to remove excess reagents and subsequently added a mixture of 3,3′,5,5′-tetramethylbenzidine and H_2_O_2_ to produce a colorimetric reaction. After 120 s, we used a smartphone camera (Apple; Cupertino, CA, USA) to capture the results, which we converted into 8-bit grayscale to determine the color intensity using image processing software (Image J; National Institutes of Health [NIH], Bethesda, MD, USA) (Figure 1).

### 2.3. Components of the Turntable Paper-Based Device

**Figure 2:**
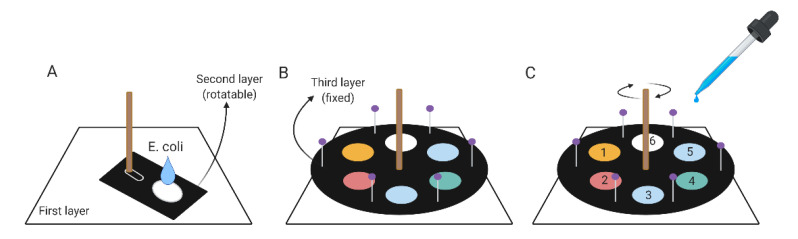
Schematic illustration of a turntable paper-based device. (**A**) *E. coli* suspensions were dropped onto the reaction zone of the rotatable second layer. (**B**) A round paper layer was fixed on top of the second layer to act as the third layer. (**C**) Reagents were added in the same order as in paper-based ELISA. Each reagent was added in specific hydrophobic zones numbering from 1 to 6: 1 for blocking buffer, 2 for anti-*E. coli* biotin conjugate antibody, 3 for washing buffer, 4 for HRP-conjugated streptavidin, 5 for washing buffer, and 6 for substrate solution. Before adding each reagent, rotation of the axis was required in order to move the reaction zone of the second layer to the correct position.

The turntable paper-based device was composed of an acrylic base at the bottom, a wooden chopstick rotation axis in the middle, and three layers of paper (Figure S1 and Figure 2). We used blotting paper for the first layer of paper because of its great absorption ability, and the second and third layers were made using Whatman^®^ Grade 3 filter paper (Sigma–Aldrich, St. Louis, MO, USA) and Whatman^®^ Grade 1 filter paper (Sigma–Aldrich; St. Louis, MO, USA), respectively. The Whatman^®^ Grade 3 filter paper (layer 2) was cut into a rectangular shape and wax-printed with a circular reacting zone (Figure 2A). This layer was fixed to the axis using a paper clip, so that it could be moved by spinning the axis. As the axis was rotated, the affixed rectangular shape could be moved, as if it was a very wide hand of a clock. The Whatman^®^ Grade 1 filter paper (layer 3) was cut into a circular, plate shape and patterned with six hydrophobic test zone areas via wax printing. This layer was fixed to the base with several pins to prevent it from spinning with the axis (Figure 2B). These pins were placed outside of the radius of the second, moving layer to prevent them from impeding the movement of the second layer. Both the second and third layers of filter paper required wax printing to create desirable reaction zones for reagent flow. Following wax printing, papers were baked in an oven at 105 °C for 5 min to form hydrophilic and hydrophobic boundaries. Bacterial suspensions were loaded onto the second layer, and reagents were loaded onto the third layer.

### 2.4. Turntable Paper-Based Device Operating Protocol

To conduct paper-based ELISA using our turntable device, a bacterial suspension was loaded onto the reaction zone of the second layer of paper, the sample was allowed to dry for an hour, and the paper was fixed to the axis with a paper clip so that it could move as the axis rotated (Figure 2A); again, like the hand of a clock. The third paper layer with wax-printed, hydrophobically bordered reaction zones was fixed on top of the second layer paper with pins to keep it from moving when the axis was rotated (Figure 2B). After assembling the device, reagents required for paper-based ELISA could be added into the hydrophobically bordered test zones of the third, top layer in a stepwise fashion, and the axis could be rotated to place the sample zone of layer 2 directly under the hydrophobically bordered reaction zone of layer 3 to conduct each step of the process (Figure 2C). During this process, reagents were added in a manner similar to the process for creating paper-based ELISA, but they were added in larger volumes to facilitate the transfer of reagent from the third layer to the second sample layer below. Aliquots of 200 μL of bacterial suspension were applied to the second layer of filter paper and allowed to rest for 1 h before being blocked with 300-μL 1% BSA, which was applied onto the adjacent third layer, and the assembly was allowed to rest for another 1 h. Rotation of the axis placed the sample zone of layer 2 directly under a reaction zone of layer 3, where 300 μL of anti-*E. coli* biotin conjugate antibody was applied before incubation for 30 min. The second layer was moved so that the sample zone of layer 2 was directly under the subsequent reaction zone of layer 3, and the aligned sample and reaction zones were washed 4 times with 300 μL of PBST buffer. After washing, the second layer was moved to the subsequent position, and 300 μL of HRP-conjugated streptavidin was applied. The second layer was moved again, and the layers were washed 4 times with PBST. The second layer was moved a final time, and 300 μL of a 3,3′,5,5′-Tetramethylbenzidine (TMB) and H_2_O_2_ mixture solution was applied to produce a colorimetric reaction. The results were photographed after 120 s using a commercially available smartphone camera, and the third layer was immediately removed. Images were converted to 8-bit grayscale with Image J to measure color intensity. This process was repeated with a new assembly for each of the seven concentrations of bacterial suspension, and the results were compared.

## 3. Results

**Figure 3:**
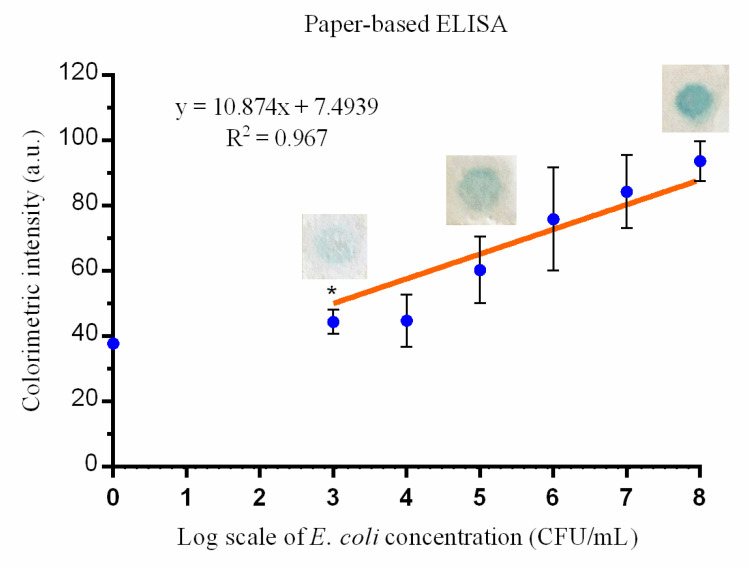
The association between the colorimetric result intensity (arbitrary units [a.u.]) from paper-based ELISA and *E. coli* inoculum (colony-forming units (CFU)/mL). The intensity difference between the lowest colony number, 10^3^ CFU/mL, and 0 CFU/mL (control) is significant (* indicates *p* < 0.05 compared with the negative control) (mean ± standard deviation (SD); results are from three independent experiments).

The results of our paper-based ELISA tests indicated that color intensity was linearly correlated with the logarithm of *E. coli* concentration, with R^2^ = 0.967 (Figure 3). The color intensity results for the *E. coli* samples with concentrations of 0 and 10^3^ CFU/mL were significantly different (*p* = 0.0355), and the limit of detection was approximately 10^3^ CFU/mL.

**Figure 4:**
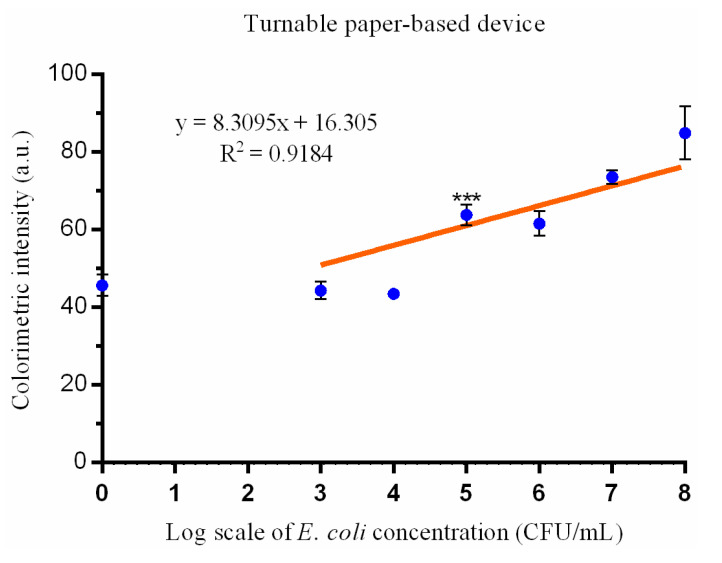
The association between the colorimetric result intensities from the turntable paper-based device and *E. coli* inoculum (CFU/mL). The intensity differences between 10^5^ CFU/mL and 0 CFU/mL (control) is significant (*** indicates *p* < 0.001 compared with the negative control) (mean ± SD; results are from three independent experiments).

The colorimetric intensity results using this turntable device and an array of *E. coli* concentrations show comparable results with those obtained from the paper-based ELISA tests. The color intensity was linearly correlated with the logarithm of the *E. coli* concentrations (R^2^ = 0.918); there was only a small standard deviation across the color intensities (Figure 4),and there was only a small standard deviation in the color intensity values (Figure 4). A significant difference in the color intensities was displayed between the control and the 10^5^ CFU/mL samples (*p* = 0.0006), and the limit of detection was approximately 10^5^ CFU/mL.

## 4. Discussion

The Surviving Sepsis Campaign guidelines note that the early initiation of appropriate antimicrobial therapy in the initial stage of sepsis can improve clinical outcomes [14]. A rapid diagnosis of the causative pathogens and identification of the potential sources of infection are crucial for selecting appropriate antimicrobial agents [14]. POC tests can help clinicians make rapid diagnoses and suitable treatment decisions [15] and have been widely used in clinical practice to diagnose infectious diseases [7,8,9]. They may also be used to provide impactful analytical results in both clinical and nonclinical settings [9]. Further, the nonclinical/home use of POC diagnostics carries the added advantages of reducing the potential transmission of contagious disease and securing personal privacy [7,9].

ELISA is a popular, easy-to-perform assay for quickly detecting antigens (within hours). This method displays a high sensitivity and specificity for quantitative antigen detection [11,16]. Several POC tests have been designed that leverage the ELISA process, including magnetic nanoparticle-based chip [17], microfluidic chip [18], lab-on-compact-disc [19], and multiplexed volumetric bar-chart chip analyses [20]. These methods, however, require complex materials that limit their use. While much of the equipment needed to conduct a conventional ELISA is inexpensive and widely available, the results are dependent on the execution of multiple steps, and quantitative results require the use of a spectrophotometer [21]. Although our previous paper-based ELISA assay [13] and others [22,23] replaced the need for a spectrophotometer with a commercially available smartphone camera, the process still requires the execution of multiple steps. In this study, we developed a turntable paper-based device for the rapid quantification of *E. coli*. The innovative design of this device allows all of the steps to be performed using a single device, and the results are in agreement with those from previously demonstrated paper-based ELISA assays.

The differences between our previously proposed paper-based ELISA and this novel turntable paper-based device are outlined in Table 1. The filter papers selected are different between the two methods. The paper used in conventional paper-based ELISA is Whatman^®^ Fusion 5 (Sigma–Aldrich; St. Louis, MO, USA), because it provides good absorption, an acceptable wicking area, optimal wicking time, and facilitates homogeneous color development [13]. However, the softness of this paper material makes the application of wax printing difficult. The structure of Fusion 5 paper is damaged after being heated and squeezed by a wax printer. The design of the turntable paper-based device requires reagents to transfer from one paper layer to another, which is different from dropping materials directly onto a single-plane reaction zone, as performed in paper-based ELISA. In order to control and restrict the absorptive flow of reagents between paper layers and promote quality, standardizable results, the application of the hydrophobic barrier wax printing that defines the sample and test zones in layers 2 and 3 is critical. We chose to use Whatman^®^ Grade 1 filter paper for layer 3, because it is one of the most widely used filter papers for routine applications and examinations [13,24,25], and it has a medium retention and flow rate [24]. We chose to use Whatman^®^ Grade 3 filter paper for the second layer because of its durability and suitability for wax printing. It is also twice as thick as Whatman^®^ Grade 1 filter paper, and it still demonstrates good absorption with a finer particle retention and excellent loading capacity. The multilayer design of this device and the necessary exchange of materials from one layer to another requires increased reagent volumes compared to a conventional paper-based ELISA. The detection limit for a paper-based ELISA was 10^3^ CFU/mL, and the detection limit for our paper-based turntable device was 10^5^ CFU/mL. Sample concentrations over the detection limit demonstrated a linear correlation against the colorimetric intensity for both approaches (R^2^ = 0.967 and R^2^ = 0.9184, respectively). R^2^, a number between 0 and 1 that explains the relationship between two variables, is a commonly reported statistic in linear models [26]. It has been widely used in laboratory analyses [27,28]. In this study, the results imply that the colorimetric intensity detected is a good predictor of the log scale value for bacterial cell concentrations. Therefore, the device could be used to quantitatively detect *E. coli*. Comparable results from both approaches indicate that this novel turntable device could be used to develop bacterial POC diagnostics. To simplify the process, optimization studies should be undertaken to select a paper substrate for the third layer that would support the immobilization of preloaded reagents.

With additional modifications, this turntable device could be especially well-suited for several applications: (1) standardizing the fabrication of each layer could facilitate the reuse of the device for detecting other pathogens via paper layer changes, and (2) fabrication optimization could result in the development of easy-to-use, at-home kits for bacterial detection.

## 5. Conclusions

We developed a turntable paper-based device to quantitatively detect the presence of *E. coli.* This method, which could simplify the conventional ELISA protocols, provided results comparable to a paper-based ELISA. Further experiments are needed to validate and enhance the device performance.

## Figures and Tables

**Table 1:** Comparison of the paper-based enzyme-linked immunosorbent assay (ELISA) and a turntable paper-based device for the detection of *Escherichia coli.* CFU: colony-forming units.

Characteristics	Paper-Based ELISA Turntable Paper-Based Device	
Filter paper	Whatman Fusion 5	Whatman Grade 3
Time	About 4 h	About 4 h
Reagents volume	200 μL	300 μL
Limit of detection	10^3^ CFU/mL	10^5^ CFU/mL

## Data Availability

The datasets generated during and/or analyzed during the current study are available from the corresponding author upon reasonable request.

## Supplementary Materials

The following are available online at https://www.mdpi.com/2072-666X/12/2/194/s1, Figure S1: Structure of turntable device. (**A**) An acrylic board drilled with a hole in the middle is the base of the whole device. (**B**) Blotting paper was placed in the first layer and was also drilled with a hole in the middle. (**C**) A chopstick which glued to a pin was stuck into the hole in the middle as the rotation axis, and the second layer filter paper was fixed to the axis by the pin. (**D**) The third layer filter paper was cut into circle shape and was penetrated by the axis in the middle. (**E**) After confirmed that the rotation of second layer is fine, fixed the third layer with base and the first layer by tacks.
