# Supplementary material for: Turntable Paper-Based Device to Detect Escherichia coli
Source: Micromachines (Basel). 2021 Feb 13;12(2):194. doi: 10.3390/mi12020194 (PMC7917795; doi:10.3390/mi12020194)
Supplement: Supplementary file 1 [file micromachines-12-00194-s001.pdf]

Article

Supplementary Materials: Turntable paper-based device to detect *Escherichia coli*

Yung-Chih Wang [1], Yao-Hung Tsai [2], Ching-Fen Shen [3], Ming-Yao He [2], Yi-Chen Fu [2], Chen-Yu Sang [2], Yi-Tzu Lee [4,5,*] and Chao-Min Cheng [2,*]

1 Division of Infectious Diseases and Tropical Medicine, Department of Internal Medicine, Tri-Service General Hospital, National Defense Medical Center, Taipei, 114, Taiwan; wystwyst@gmail.com
2 Institute of Biomedical Engineering, National Tsing Hua University, Hsinchu, 300, Taiwan; michaeltsai45@gmail.com (Y.-H.T.); martin880210@gmail.com (M.-Y.H.); sandy216621@gmail.com (Y.-C.F.); sang0205@gmail.com (C.-Y.S.)
3 Department of Pediatrics, National Cheng Kung University Hospital, College of Medicine, National Cheng Kung University, Tainan, 701,Taiwan; drshen1112@gmail.com
4 Department of Emergency Medicine, Taipei Veterans General Hospital, Taipei, 112, Taiwan
5 Faculty of Medicine, School of Medicine, National Yang-Ming University, Taipei, 112, Taiwan
* Correspondence: chaomin@mx.nthu.edu.tw (C.-M.C.); s851009@yahoo.com.tw (Y.-T. L.)

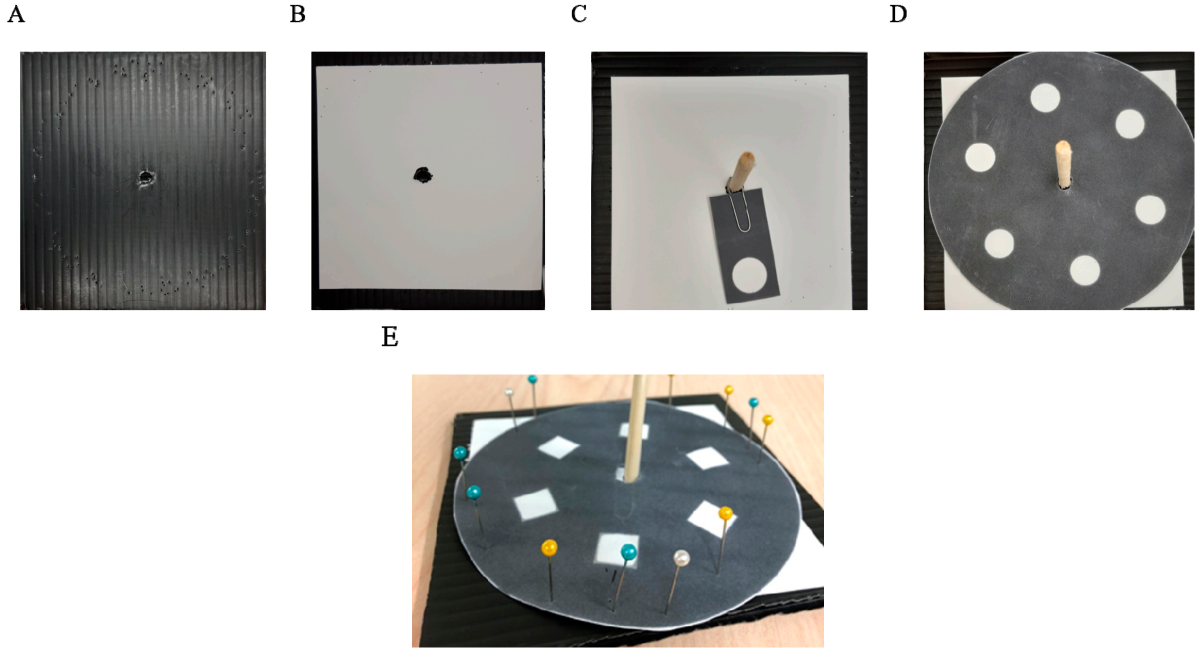

Figure S1. Structure of turntable device. (**A**) An acrylic board drilled with a hole in the middle is the base of the whole device. (**B**) Blotting paper was placed in the first layer and was also drilled with a hole in the middle. (**C**) A chopstick which glued to a pin was stuck into the hole in the middle as the rotation axis, and the second layer filter paper was fixed to the axis by the pin. (**D**) The third layer filter paper was cut into circle shape and was penetrated by the axis in the middle. (**E**) After confirmed that the rotation of second layer is fine, fixed the third layer with base and the first layer by tacks.
